# Comparative Transcriptome Analysis of *Pseudomonas putida* KT2440 Revealed Its Response Mechanisms to Elevated Levels of Zinc Stress

**DOI:** 10.3389/fmicb.2018.01669

**Published:** 2018-07-24

**Authors:** Jun Peng, Lihong Miao, Xi Chen, Pulin Liu

**Affiliations:** ^1^College of Biological and Pharmaceutical Engineering, Wuhan Polytechnic University, Wuhan, China; ^2^Wuhan Institute of Virology, Chinese Academy of Science, Wuhan, China

**Keywords:** *Pseudomonas putida*, zinc, transcriptome analysis, stress response, metal transport, membrane homeostasis, oxidative stress, basic metabolism

## Abstract

The whole-genome transcriptional response of *Pseudomonas putida* KT2440 to stress-inducing concentrations of zinc was analyzed in this study by RNA sequencing to thoroughly investigate the bacterial cell response to zinc toxicity. The data revealed that different levels of zinc stress strongly affected the transcription of genes from the following categories: metal transport genes, genes involved in membrane homeostasis, oxidative-stress-responding genes, and genes associated with basic cellular metabolism. At the lowest zinc dose, only several genes associated with metal transport and membrane homeostasis were strongly influenced. At the intermediate zinc dose, transcriptional changes of genes belonging to these two categories were highly pronounced. In addition, the intermediate zinc stress produced high levels of oxidative stress, and influenced amino acid metabolism and respiratory chains of *P. putida*. At the highest zinc dose, the induction of genes responsible for Fe–S cluster biogenesis was the most remarkable feature. Moreover, upregulation of glyoxylate cycle was observed. In summary, the adaptation of the cell envelope, the maintenance of metal homeostasis and intracellular redox status, and the transcriptional control of metabolism are the main elements of stress response, which facilitates the survival of *P. putida* KT2440 in zinc-polluted environments.

## Introduction

Zn^2+^ is an essential transition metal ion that plays important roles in enzyme catalysis, protein structure, and transcriptional regulation in organisms from all three kingdoms (Blencowe and Morby, 2003). However, excess zinc is toxic to cells in a variety of ways, such as replacing other metal ions from enzymes, modifying stability of biomolecules, and damaging cells' antioxidant defense systems (Hobman et al., 2007; Nejdl et al., 2014; Krezel and Maret, 2016). This dual behavior has made developing resistance systems necessary for organisms to survive in zinc-polluted environments.

Metal resistance systems in bacteria are abundant and widespread (Silver and Phung, 2005; Barnett et al., 2012). Maintaining low levels of cytosolic metal ions is a key strategy to withstand metal toxicity (Aguilar-Barajas et al., 2010). Bacteria are devoid of subcellular compartments, and the main mechanisms that control cellular zinc concentration are limited to the precisely regulated processes of zinc influx, efflux, and sequestration (Blencowe and Morby, 2003; Gadd, 2010). Zinc can be transported into bacterial cells via ZnuACB, a high-affinity Zn^2+^ uptake system (Ammendola et al., 2007; Bhubhanil et al., 2014; Tanaka et al., 2018), and some other broad-spectrum metal ion transporters or channels, such as HmtA (Gonzalez et al., 2018), MntABC (Tanaka et al., 2018) and Pit (Beard et al., 2008). Once the intracellular zinc is present above a certain threshold concentration, excess zinc is effluxed from the cytoplasm or periplasmic space by members of cation diffusion family, resistance–nodulation–cell division family, and P-type ATPase (Nies, 2007). Extracellular sequestration is an important mechanism in immobilizing effluxed metal ions to prevent their re-entry (Taghavi et al., 2009). Numerous bacteria produce metabolites that result in precipitation of metals, such as phosphate, sulfur, and siderophores (Etesami, 2018). Furthermore, intracellular sequestration may be involved in zinc homeostasis in some bacteria, since several metallothionein-like proteins were identified and purified, which bind multiple zinc ions with high stability toward protons (Blindauer et al., 2002).

In recent years, genome-wide transcriptional analyses of *Escherichia coli* and *Bacillus subtilis* in metal ion stress have strongly implied that it is not only the metal-transport genes that confer resistance to the metal, but also the activation of several different stress response systems. According to microarray analysis, zinc does not significantly induce oxidative stress responses in *E. coli*; however, three important membrane stress-related regulons, *cpxRA, rpoE*, and *basRS*, are activated (Lee et al., 2005; Yamamoto and Ishihama, 2005; Hobman et al., 2007). In non-pathogenic *E. coli*, activated CpxR increases the production of membrane chaperons and protease, which alleviates periplasmic stress (Dbeibo et al., 2018), whereas *basRS* controls the biogenesis of capsular- and lipo-polysaccharides (Hobman et al., 2007). RpoE-mediated signaling pathway is potently activated by outer membrane protein assembly defects (Barchinger and Ades, 2013). The major role of *rpoE* regulon is considered to be restoring outer membrane protein folding by inducing chaperon production to maintain nascent outer membrane proteins in folding-competent states and by increasing the expression of β-barrel assembly machinery (Grabowicz and Silhavy, 2017). Furthermore, the amino acid metabolism pathways are also fine-tuned during zinc exposure. For example, both *E. coli* and *B. subtilis* upregulate the syntheses of cysteine and histidine after zinc treatment (Hobman et al., 2007).

*Pseudomonas putida* is a metabolically versatile saprophytic bacterium with great adaptability to diverse environments (Cánovas et al., 2003). Although the genomic sequence data deposited in Genbank allow for the listing of putative gene involved in zinc homeostasis, they do not provide functional evidence. Recently, Mumm et al. (2016) detected the proteomic responses of *P. putida* PaW85 to zinc excess by inoculating it into LB medium containing 0.6 mmol L^−1^ ZnSO4. However, cellular responses of *P. putida* to different levels of zinc stress remain poorly understood. To obtain insights into the dose-dependent transcriptional responses of *P. putida* to external zinc, the transcriptomes of *P. putida* KT2440 were sequenced in this study. Our results revealed that different sets of genes were activated or repressed in response to elevated levels of zinc ions. These results provide in-depth understanding of the adaptation mechanisms used by *P. putida* to survive in zinc-contaminated environments.

## Materials and methods

### Bacterial strains and culture conditions

*P. putida* KT2440 were routinely grown in LB medium at 30°C. Before being used as inoculates, they were cultured for 30 h to reach the stationary phase. A semi-synthetic cation-defined medium (CDM) (Pederick et al., 2015) was used to study the effect of zinc exposure. The exponential phased cells (about 1.0 × 10^7^ CFU mL^−1^) were treated with different zinc sulfate concentrations and incubated at 30°C. At intervals, aliquots of control and treated cultures were diluted and plated on CDM agar plates. The plates were incubated at 30°C for 40 h, and the viable cells were counted. The growth inhibition of *P. putida* KT2440 was calculated according to the following equation: growth inhibition = (cell concentration of control samples – cell concentration of zinc treated samples)/cell concentration of control samples × 100%.

Cultivating *P. putida* KT2440 for studying the gene transcription under zinc stress was done by first inoculating 100 mL CDM medium with 1 mL stationary *P. putida* culture. This culture was also allowed to grow to a cell density of 1.0 × 10^7^ CFU mL^−1^. Afterward, the culture was divided into equal portions in glass tubes. One tube was used as control, whereas the others were challenged with 0.2, 1.5, and 2.5 mmol L^−1^ zinc sulfate, respectively. After zinc treatment for 1 h (about half a generation time), each subculture was immediately collected, and the cells were harvested by centrifugation (8,000 g, 1 min). The pellets were stabilized by using RNAprotect bacteria reagent (Qiagen, Valencia, CA, USA) and then stored at −80°C before RNA extraction. Three biological replicates were performed for each treatment.

### RNA-seq analysis

Total RNAs were extracted from each sample using Trizol reagent (Invitrogen, Carlsbad, CA, USA) and treated by DNAase I. Ribosomal RNA was removed from the total RNA samples using the MICROB*Express* bacterial mRNA enrichment kit (Ambion, Austin, Texas, USA) according to manufacturer's protocol. Total and messenger RNA quantities and quality were assessed by Nanodrop spectrophotometer and electrophoresis on a 1% denaturing agarose gel. cDNA library preparation and RNA sequencing was performed by Beijing Genomics Institute (Beijing, China). Libraries were constructed using the TruSeq stranded mRNA-seq sample preparation kit (Illumina, San Diego, CA, USA), and sequencing was performed with an Illumina Hiseq 2500 platform (Illumina, San Diego, CA, USA) in rapid mode with 150 nt read length.

Raw data from Illumina sequencing platform were trimmed using Skewer version 0.2.2 (Jiang et al., 2014). Quality control was performed using Fast QC version 0.11.5 (Babraham Institute, Babraham, Cambridgeshire, UK). The (i) short reads (<20 nucleotides), (ii) adapter-dimer reads, (iii) reads with an N ratio (the number of unknown nucleotides/the number of total nucleotides) of greater than 5%, and (iv) reads with more than 20% low-quality nucleotides (Phred quality score < 10) were removed. Rockhopper 2 (McClure et al., 2013; Tjaden, 2015) was then used to align the remaining reads to the *P. putida* KT2440 genome (Winsor et al., 2016) and calculated the level of gene expression. Adjusted *P*-values (*q*-values < 0.01) were used for controlling the false discovery rate. Global gene transcription similarities/dissimilarities among samples were examined by non-metric multidimensional scaling (nMDS) (SPSS 20.0, Chicago, IL, USA).

### Validation of RNA-seq results by RT-qPCR

Reverse transcription quantitative PCR (RT-qPCR) was used to validate the RNA-seq results. A total of 15 genes were used for validation. The gene-specific primers used in this study are shown in Table S1, and their specificity was confirmed by melting curve analysis. *rsd/algQ* (the gene coding the regulator of sigma factor RpoD) was used as the internal reference because it has high transcriptional stability under polymetallic stressed conditions (Gómez-Sagasti et al., 2015). RT-qPCR analyses were performed with the same RNA samples used for RNAseq analysis. For cDNA synthesis, 1 μg of RNA was reverse transcribed using PrimeScript RT reagent kit (Takara, Dalian, China). The cDNAs were quantitatively analyzed with a Bio-Rad iCycler machine (Bio-Rad, Berkeley, CA, USA) using Sybr Green. The following program was used: 95°C for 1 min, followed by 40 cycles of 95°C for 10 s, 60°C for 15 s, and 72°C for 15 s. Melting temperature-determining dissociation steps were performed at 95°C for 15 s, 60°C for 30 s, and 95°C for 15 s. The relative expression ratio was calculated as the relative quantity of the target gene transcript under zinc treatment conditions divided by the relative quantity of the target gene transcript under control conditions. Normality tests (Shapiro–Wilk) were conducted for the fold-change data of RNA-seq and RT-qPCR. As all groups followed the normal distribution, Pearson's correlation coefficient was calculated to determine the level of association among variables (SPSS 20.0, Chicago, IL, USA).

## Results and discussion

### Effect of sublethal concentrations of zinc on *P. putida* KT2440 growth

**Figure 1:**
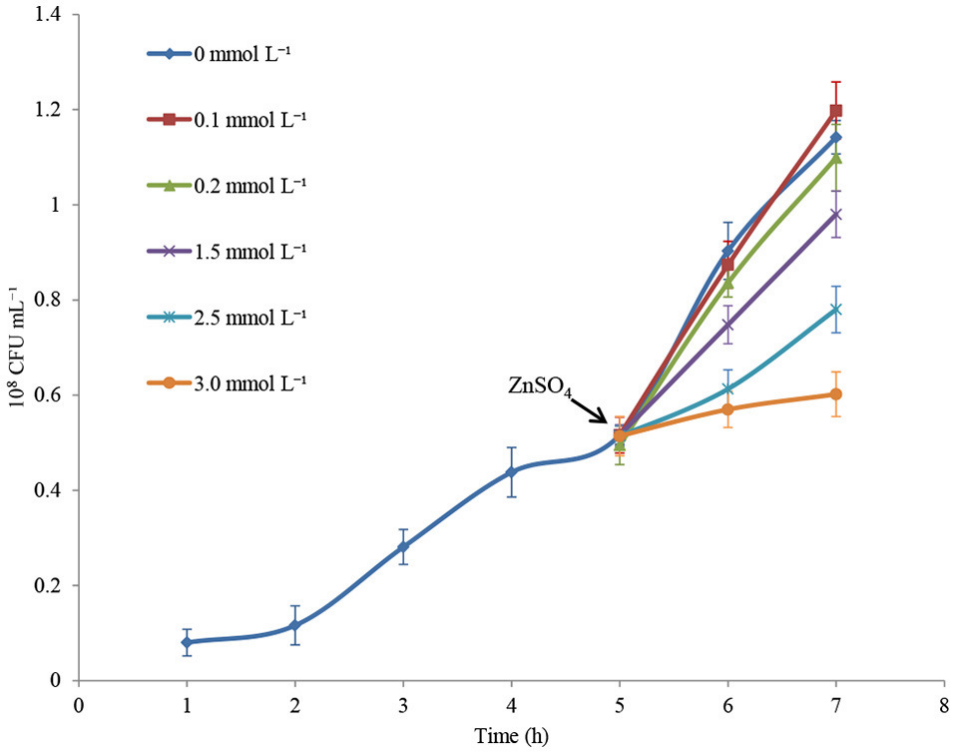
Growth curves of *P. putida* KT2440 cultured in CDM medium or in CDM medium supplemented with zinc. Addition of zinc is indicated by arrow. The values are means for triplicate cultures. The error bars indicate standard deviations.

Cell growth in CDM medium was monitored for 7 h, and changes in cell concentration following treatment with increasing concentrations of ZnSO_4_ were measured. Figure 1 shows that zinc sulfate concentration of 0.1 mmol L^−1^ did not affect the specific growth rate. Conversely, 3.0 mmol L^−1^ zinc sulfate almost completely inhibited the growth of *P. putida* KT2440. When 0.2, 1.5, and 2.5 mmol L^−1^ zinc were present, about 5%, 40%, and 80% inhibition rates of cell growth were observed in the 6-h-cultured samples. In the following RNAseq analysis, 0.2, 1.5, and 2.5 mmol L^−1^ of zinc sulfate were used to determine the transcriptional responses of *P. putida* KT2440 to different levels of zinc stress.

### Transcriptome features under zinc treatment

**Figure 2:**
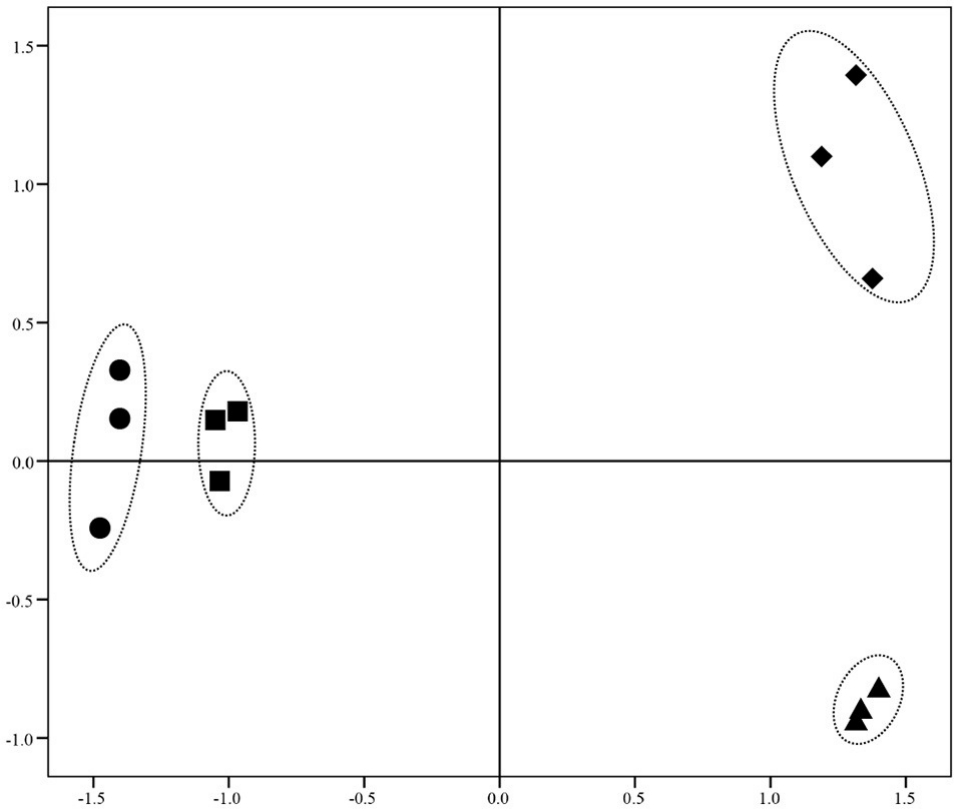
nMDS analysis of zinc treated samples via their transcriptional profiles. Increasing distance between points on the nMDS plot equates to the increasing dissimilarity in transcriptional profiles. The symbols •, ■, ▴, and ♦ represent transcriptional profiles obtained from the samples treated with 0, 0.2, 1.5, and 2.5 mmol L^−1^ zinc, respectively.

**Figure 3:**
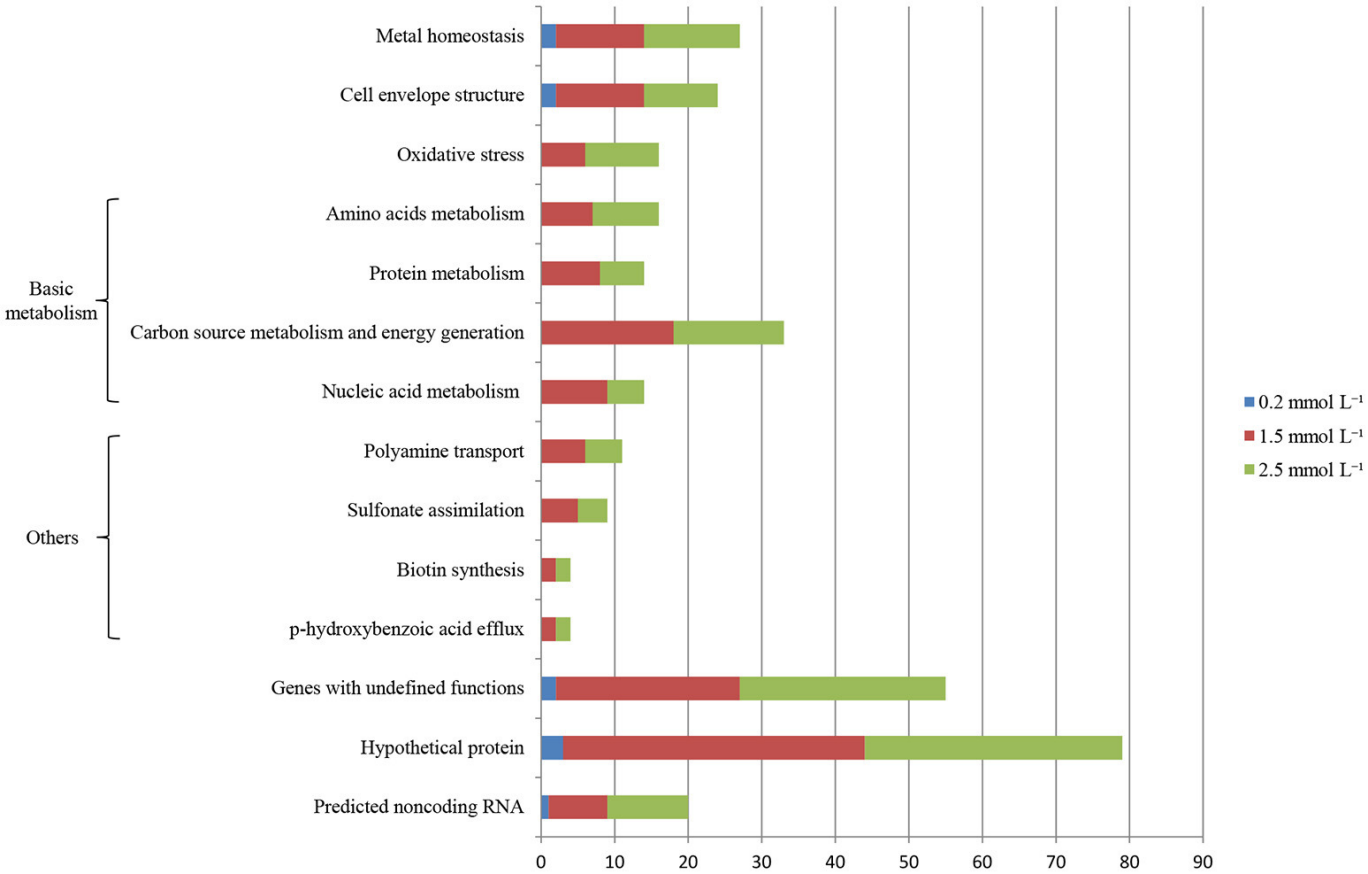
Distribution of genes whose transcription levels were strongly affected by zinc treatment. The top 11 functional categories affected by zinc are illustrated. Genes involved in polyamine transport, *p*-hydroxybenzoic acid efflux, biotin synthesis, and sulfonate assimilation are sorted into an artificial group designed as “others”.

Illumina Hiseq 2500 platform produced an average read length of about 170 bp. The number of reads obtained for each sample ranged from 11.3 to 15.8 million (Table S2), with about 83.4% mapping the *P. putida* KT2440 genome. To obtain an overview of changes in gene transcriptions elicited by zinc stress, nMDS analysis was performed to visualize the total similarity of the different transcription profiles. In the nMDS plot, distance indicated the unique association of different samples, and the samples that appeared close were those with close proximity (Liu et al., 2017). As shown in Figure 2, there was greater variability between the different experimental conditions than within each biological replicate group. Smaller differences in overall gene transcription profiles between the 0.2 mmol L^−1^ zinc treated and the control samples were observed. Zinc treatment at two higher concentrations led to transcription profiles that deviated significantly from that of the untreated cells. Overall, 849 genes (14.77% of the genome) were influenced by more than two-fold in at least one condition, and 213 genes were strongly up- or downregulated by at least 4-fold. As expected, 0.2 mmol L^−1^ zinc affected a relatively small set of genes under these treatment conditions. 2.5 mmol L^−1^ zinc had the greatest effect on the cell, with a 4-fold or greater upregulation of 125 (2.17% of the genome) and downregulation of 29 genes (0.50% of the genome) (Table 1). Analysis of the strongly influenced genes with known functions revealed that all three levels of zinc stress mainly affected the transcription of genes from the following four categories (Figure 3): metal homeostasis genes (7.51%), genes involved in cell envelope structure (6.57%), antioxidant enzyme encoding genes (6.57%), and genes associated with basic cellular metabolism (24.41%). The functions of hypothetical proteins and newly identified noncoding RNAs (Table S3) are poorly understood, their roles in zinc homeostasis are not discussed in this study.

**Table 1:** Summary of transcriptome data^a^.

**Zinc concentration (mmol L^−1^)**	**Upregulate**	**Downregulate**	**Combined**	**%Genome^b^**
0.2	3 (27)	2 (9)	5 (36)	0.08 (0.63)
1.5	92 (311)	38 (284)	130 (595)	2.28 (10.35)
2.5	125 (434)	29 (156)	154 (590)	2.68 (10.26)

a Number of genes with changes greater than four (and 2-fold in parentheses) after zinc addition.

b *Percentage of the 5,748 protein encoding genes (Winsor et al., 2016) with changes more than 4- (2-) fold*.

### Genes involved in metal transport

Controlling zinc ions transport is the most effective detoxification mechanism employed by bacteria to cope with zinc stress. Genome analysis has revealed that *P. putida* KT2440 evolved an unexpectedly large variety of genes involved in metal homeostasis, among which about 40% of zinc-transport related genes appeared to be clustered into an 8 kb region around the chromosome replication origin, including *cadRA*1, *czcDRS*1, *czcCBA*1, and *znuABC*1 (Cánovas et al., 2003; Wu et al., 2011). For the zinc-transport related genes located far from the *ori* region, most of them seem to be generated by duplication, such as *cadRA*2, *czcDRS*2, *czcCBA*2, and *znuABC*2. Gene duplication is a major mechanism through which genes with new functions are generated during evolution (Serres et al., 2009; Kondrashov, 2012; Katju and Bergthorsson, 2013). The benefits of gene duplication in metal resistance can be clearly seen in *Cupriavidus metallidurans* (von Rozycki and Nies, 2009). The transcription of *cadA*1, *cadA*2, *czcCBA*1, and *czcCBA*2 in *P. putida* KT2440 has been analyzed by Leedjärv et al. (2008) after cloning their promoter regions into reporter plasmids. In accordance with their report, only *cadA*1, *cadA*2, and *czcCBA*1 responded to increased zinc ions in this study (Table 2). Moreover, *cadA*2 was induced in all the three zinc treatment conditions. Under the intermediate level of zinc stress, *cadA*1 and *czcA*1 were upregulated by 9.57-fold and 49.33-fold, respectively; in addition, the transcription of *czcD* was significantly upregulated (fold change = 9.20). Different inducibilities were also observed among some of the other duplicated genes, such as *czcRS*, the two component system that has been proven to be involved in the induction of *czcCBA* in *C. metallidutans* and *P. aeruginosa* (Große et al., 2004; Perron et al., 2004) with the presence of high levels of several divalent metal ions. The two complete duplications of *czcRS* in *P. putida* KT2440 appeared to be sub-functionalized. *czcR*1 was constitutely transcripted in the control samples (Expression value = 234, calculated by Rockhopper), which may act as a readily available metal-sensing system to cope with sudden increased metal stress. *czcR*2 and *czcR*3 positively responded to 1.5 mmol L^−1^ zinc, which increased their transcription by 37.78-fold and 27.50-fold, respectively. As the zinc concentrations increased to 2.5 mmol L^−1^, a slight decrease was observed in the transcription of these zinc-related efflux systems compared with that in the 1.5 mmol L^−1^ zinc treatment. However, *nik* operon (*nik*A, *nik*B, and *nik*E), which encodes a nickel-import system, and four genes encoding arsenate resistance systems were significantly upregulated. Zinc can replace Ni ions from their enzymes (Aguilar-Barajas et al., 2010); therefore, nickel uptake ATPase was reasonably upregulated to counter the Ni defect induced by zinc toxicity. *P. putida* KT2440 evolved two *asr* operons, *arsRBCH*1 and *arsRBCH*2, both of them appeared to be upregulated, which was also confirmed by RT-PCR. Arsenic resistance is mainly mediated by the *arsRBC* genes, which is usually found in most bacteria (Cánovas et al., 2003). *arsC* encodes an arsenic reductase that transform As(V) into As(III); ArsB is a secondary transporter responsible for the extrusion of As(III), Sb(III), or Bi(III) using the proton motive force; *arsR* encodes a transcription regulator that de-represses *arsBC* expression (Cánovas et al., 2003; Moore and Gaballa, 2005). As arsenic and zinc share little similarity, it hard to believe that *arsC* also effluxing zinc out of the cells. We inferred that the upregulation of these two operons was caused by the unspecific induction of the *arsR*. In *B. subtilis* the transcription of *arsR* was not only induced by As(III) and Bi(III), but also strongly by Cd(II) and Ag(I) (Moore and Gaballa, 2005).

**Table 2:** Expression ratios of genes involved in metal transport^a^.

***P. putida* KT2440 ORF^b^**	**Gene name**	**Fold change**^**c**^			**Annotation**
		**0.2 mmol L^−1^**	**1.5 mmol L^−1^**	**2.5 mmol L^−1^**	
PP_0026	*czcD*	–^d^	**9.2** ± **0.40**	**6.40** ± **1.04**	Cobalt/cadmium/zinc exporter
PP_0041	*cadA*1	–	**9.57** ± **1.90**	**4.26** ± **0.05**	Cadmium translocating P-type ATPase
PP_0043	*czcA*1	3.16 ± 0.47	**49.33** ± **1.89**	**22.67** ± **4.54**	Cation efflux system protein
PP_0044	*czcB*1	–	**34.70** ± **3.81**	**13.9** ± **3.09**	Cobalt/zinc/cadmium resistance protein
PP_0045	*czcC*1	–	**49.40** ± **5.27**	**16.60** ± **2.02**	Cobalt/zinc/cadmium resistance protein
PP_0047	*czcR*3	–	**27.50** ± **4.19**	**15.00** ± **4.16**	Response regulator
PP_1437	*czcS*2	–	**14.27** ± **0.49**	**13.27** ± **1.38**	Heavy metal sensor histidine kinase
PP_1438	*czcR*2	–	**37.78** ± **1.49**	**32.78** ± **0.32**	Response regulator
PP_1929	*arsB*1	–	3.0 ± 0.42	**8.5** ± **1.84**	Arsenite/antimonite transporter
PP_1930	*arsR*1	–	**5.22** ± **0.04**	**15.00** ± **3.89**	Arsenic resistance transcriptional regulator
PP_2716	*arsC*2	–	–	**5.14** ± **1.45**	Arsenate reductase
PP_2718	*arsR*2	–	3.67 ± 0.94	**18.00** ± **4.71**	Arsenic resistance transcriptional regulator
PP_3342	*nikA*		**4.25** ± **1.00**	2.5 ± 0.18	Nikel ABC transporter
PP_3343	*nikB*		**5.50** ± **0.54**	3.0 ± 1.13	Nikel ABC transporter
PP_3346	*nik*E	–	3.43 ± 0.40	2.29 ± 0.23	Nikel ABC transporter ATP-binding protein
PP_5139	*cadA*2	**5.00** ± **0.67**	**114.22** ± **11.37**	**159.33** ± **14.09**	Cadmium translocating P-type ATPase

a Expression levels of genes in P. putida KT2440 were measured using RNA-seq, as described in section Materials and methods. The data are mean ± standard deviation of three replicates. If a gene was strongly changed more than 4-fold at one experimental condition, the changes at other conditions are also shown.

b Gene name, number, and annotation are from the Pseudomonas genome project (Winsor et al., 2016).

c Fold change ≥4 or ≤ 0.25 was illustrated in bold.

d *Dashes mean that the transcription was not significantly changed (Fold change ≤2 or ≥0.5, or P_adj_ > 0.01)*.

### Genes involved in cell envelope homeostasis

The bacterial cell envelope comprises the inner cell membrane and/or the cell wall, which provides structural integrity to the cell, and acts as a sensory interface and molecular sieve mediating both the controlled solute transportation and information flow (Jordan et al., 2008). Maintaining the cellular envelope balance in the presence of stress conditions is crucial for the bacteria's survival. Previous studies have demonstrated that zinc ions act as a strong envelope-perturbing agent in *E. coli* (Hobman et al., 2007). Moreover, zinc ions induce membrane stress responses in *P. aeruginosa* PA14 and *P. putida* PaW85 as the ColRS two-component signal transduction system is activated (Nowicki et al., 2015; Mumm et al., 2016). In this study, 1.5 mmol L^−1^ zinc induced the transcription of *dgkA*1 (fold change = 4.67), which is responsible for the glycerophospholipid biosynthesis, and *plpB* (fold change = 4.90), whose product is NlpA lipoprotein (Table 3). In *E. coli*, NlpA lipoprotein is an inner membrane protein contributing to the biogenesis of outer membrane vesicles (Bodero et al., 2007). The induction of NlpA in *P. putida* suggests that the formation of outer membrane vesicles was enhanced. Accelerating the formation of outer membrane vesicles was proven to be an adaptive response of *P. putida* to several kinds of environmental stress, such as heat shock, high NaCl or EDTA concentration (Baumgarten et al., 2012). The released outer membrane vesicles lead to an increased cell surface hydrophobicity as well as to a higher tendency to form biofilms (Baumgarten et al., 2012).

**Table 3:** Expression ratios of genes involved in cell envelope homeostasis^a^.

***P. putida* KT2440 ORF^b^**	**Gene name**	**Fold change**^**c**^			**Annotation**
		**0.2 mmol L^−1^**	**1.5 mmol L^−1^**	**2.5 mmol L^−1^**	
PP_0033		–^d^	**54.20** ± **4.73**	**16.20** ± **5.11**	Undecaprenyl-glycosyl transferase
PP_0035		–	**127.50** ± **5.30**	**40.00** ± **4.95**	Bactoprenol-linked glycose transferase
PP_0037	*oprP*	–	**13.25** ± **4.15**	3.25 ± 1.22	Porin P
PP_0046	*opdT*1	**29.75** ± **5.77**	**96.75** ± **5.35**	**49.00** ± **5.32**	Tyrosine-specific outer membrane porin D
PP_0268	*oprQ*	–	**4.67** ± **0.33**	**8.89** ± **0.64**	Outer-membrane porin D
PP_0799	*opdC*	–	3.36 ± 0.15	**6.86** ± **1.01**	Histidine-specific outer membrane porin D
PP_0904		**0.17** ± **0.01**	3.57 ± 0.24	3.91 ± 0.07	Lipopolysaccharide kinase
PP_1019	*oprB*1	–	**0.20** ± **0.01**	**0.20** ± **0.01**	Carbohydrate-selective porin
PP_1121		–	**0.24** ± **0.00**	**0.23** ± **0.04**	OmpA family protein
PP_1206	*oprD*	–	**0.23** ± **0.03**	**0.26** ± **0.03**	Basic amino acid specific porin OprD
PP_1636	*dgkA*1	–	**4.67** ± **0.21**	3.70 ± 0.31	diacylglycerol kinase
PP_3764	*opdN*	–	**8.25** ± **0.35**	**12.75** ± **1.24**	Outer-membrane porin D
PP_4282	*aqpZ*	–	**0.23** ± **0.00**	0.36 ± 0.04	Aquaporin Z
PP_5165	*plpB*	–	**4.90** ± **0.04**	3.90 ± 0.01	NlpA lipoprotein

a Expression levels of genes in P. putida KT2440 were measured using RNA-seq, as described in section Materials and methods. The data are mean ± standard deviation of three replicates. If a gene was strongly changed more than 4-fold at one experimental condition, the changes at other conditions are also shown.

b Gene name, number, and annotation are from the Pseudomonas genome project (Winsor et al., 2016).

c Fold change ≥4 or ≤ 0.25 was illustrated in bold.

d *Dashes mean that the transcription was not significantly changed (Fold change ≤ 2 or ≥0.5, or P_adj_ > 0.01)*.

Unlike the plasma membrane, the outer membrane of gram-negative bacteria is relatively permeable to small molecules. A group of proteins, known as porins, form fluid-filled channels in the outer membrane that allow hydrophilic solute to diffuse across in to the periplasmic space (Blencowe and Morby, 2003). The exposure of *P. putida* KT2440 to zinc greatly altered the transcription of several porin encoding genes. For example, the *Pseudomonads* phosphate-specific porin, OprP (Pongprayoon et al., 2009), was upregulated by 13.25-fold under intermediate zinc stress. The zinc-specific induction of *oprP* may explain the need to enhance the phospholipid biosynthesis and maintain the membrane integrity. Two porins that facilitated the diffusion of tyrosine (OpdT1) and histidine (OpdC, only induced by 2.5 mmol L^−1^ zinc) were also upregulated. *oprD* was commonly downregulated by 1.5 mmol L^−1^ (fold change = 0.23) and 2.5 mmol L^−1^ (fold change = 0.26) zinc treatment in this study. In *P. aeruginosa*, the transcription of *oprD* was reduced by copper and zinc (Caille et al., 2007). Nevertheless, OprD was not directly involved in heavy metal resistance because an *oprD* knock-out mutant was as susceptible as the wild-type to heavy metals (Perron et al., 2004). CzcRS is a regulatory system that connects *czcCBA* and *oprD* expression; unphosphorylated CzcR repress the transcription *oprD*, whereas the amount of active phosphorylated CzcR is critical for inducing *czcCBA* (Perron et al., 2004). Therefore, *oprD* downregulation is a secondary effect linked to *czcCBA* overtranscription. Another porin commonly downregulated under the intermedium and high zinc stress is a carbohydrate-specific outer membrane porin, OprB1. The repression of *oprB*1 appeared to contract with the requirement of more energy because much cellular damage must be restored. Other channels were possibly used to compensate for its downregulation.

### Oxidative stress responding genes

A number of studies performed on biological systems have shown that redox-active metals can undergo redox cycling reactions and produce reactive oxygen species (ROS) (Jomova et al., 2012). Although many transition metal ions have no redox activity under physiological conditions, they can cause oxidative responses by damaging cell's antioxidant defense systems (Hobman et al., 2007). Alhasawi et al. (2014) confirmed that zinc toxicity led to oxidative stress in *P. fluorescence*; a twofold increase in oxidized protein was observed in the zinc-challenged cells compared with the control. Our transcriptomic data showed that low level of zinc stress did not enhance the transcription of antioxidant enzyme encoding genes. However, alkylhydroperoxide reductase was upregulated with increasing zinc concentration (Table 4). This thilol-specific peroxidase catalyzes the reduction of hydrogen peroxide and organic hydroperoxides to their respective alcohols (Harris, 2003). The induction of ferredoxin-NADP reductase was observed under the 1.5 mmol L^−1^ zinc treatment, which is critical for the maintenance of appropriate levels of NADPH (Ray et al., 2013). Compared with the bacteria exposed to the intermediate zinc dose, the upregulation of *isu* operon responsible for Fe–S cluster biogenesis was the most remarkable feature of the *P. putida* cells under high zinc stress. The *isu* operon is under the transcriptional control of the IscR repressor, which contains three cysteine residues and is shown to be an Fe–S protein (Schwartz et al., 2001). Holo-IscR is able to repress transcription of the *isu* operon. When Fe–S cluster assembly is disturbed by ROS or iron limitation, apo-IscR predominates, and *isc* expression increases to meet the demand (Ayala-Castro et al., 2008). Directly replacing Fe from the Fe–S clusters is another way for zinc to perturb the function of Fe–S clusters (Xu and Imlay, 2012), which also increases the amount of free Fe ions to participate in Fenton reaction. Therefore, zinc-induced oxidative stress is further enhanced.

**Table 4:** Expression ratios of oxidative-responding genes^a^.

***P. putida* KT2440 ORF^b^**	**Gene name**	**Fold change**^**c**^			**Annotation**
		**0.2 mmol L^−1^**	**1.5 mmol L^−1^**	**2.5 mmol L^−1^**	
PP_0206		–^d^	**7.50** ± **0.01**	**8.50** ± **2.47**	Ferredoxin
PP_0841	*iscR*	–	2.22 ± 0.04	–	DNA-binding transcriptional regulator IscR
PP_0842	*iscS*1		2.42 ± 0.06	**5.01** ± **1.82**	Cysteine desulfurase
PP_0843	*iscU*	–	–	**5.07** ± **1.30**	Iron-sulfur cluster assembly scaffold protein
PP_0844	*iscA*	–	2.43 ± 0.16	**4.97** ± **1.36**	Copper(I)binding iron-sulfur cluster assembly protein
PP_0845	*hscB*	–	2.35 ± 0.03	**4.59** ± **0.98**	DnaJ-like molecular chaperon
PP_0847	*fdx*	–	2.44 ± 0.10	**4.71** ± **0.45**	Ferredoxin
PP_1638	*fpr*1	–	**4.98** ± **0.23**	3.49 ± 0.11	Ferredoxin-NADP(+) rductase
PP_2023		–	2.41 ± 0.12	**6.14** ± **1.14**	Glutathione S-ttransferase family protein
PP_2439	*ahpC*	–	**6.50** ± **0.27**	**13.31** ± **0.34**	Peroxiredoxin/alkyhydroperoxide reductase small subunit
PP_2440	*ahpF*	–	**4.83** ± **0.54**	**8.67** ± **0.59**	Alkyhydroperoxide reductase sunbunit F
PP_3639		–	**4.00** ± **1.63**	3.00 ± 1.06	Alkylhydroperoxidase AphD domain-containing protein

a Expression levels of genes in P. putida KT2440 were measured using RNA-seq, as described in section Materials and methods. The data are mean ± standard deviation of three replicates. If a gene was strongly changed more than 4-fold at one experimental condition, the changes at other conditions are also shown.

b Gene name, number, and annotation are from the Pseudomonas genome project (Winsor et al., 2016).

c Fold change ≥4 or ≤ 0.25 was illustrated in bold.

d *Dashes mean that the transcription was not significantly changed (Fold change ≤ 2 or ≥0.5, or P_adj_ > 0.01)*.

### Genes involved in basic cellular metabolism

Among all the genes whose transcription was strongly influenced, about 24.41% of them were involved in basic cellular metabolism. Of these, 11 amino acid metabolism genes were up or downregulated by more than 4-fold after zinc treatment (Table 5). The synthesis of glutamate was enhanced, as revealed by the great upregulation of glutamate synthase (PP_0269). The transcription of *hutU* and PP_1110 were strongly increased under the high zinc stress. HutU is involved in the second step of the subway that synthesizes L-glutamate from L-histidine, whereas PP_1110 encodes serine acetyltransferase which catalyzes the first step that convert L-serine to L-cysteine. Glutamate and cysteine are two substrates for glutathione synthesis, and their alteration could reflect increased synthesis of glutathione. Moreover, glutamate was also shown to play an important role in the bacteria adaption to noxious conditions caused by Ni (Ray et al., 2013). Among all the up-regulated genes associated with amino acid metabolism, the most up-regulated gene was *aruC* which encodes an acetylornithine aminotransferase. In *P. aeruginosa*, this enzyme palys dual roles in arginine metabolism, which catalyze the transamination of L-glutamate with *N*-2-acetyl-L-glutamate 5-semialdehyde when functioning in arginine biosynthesis and transaminates L-ornithine with 2-oxoglutarate when participating in L-arginine degradation (Voellmy and Leisinger, 1975; Schomburg et al., 2008). Therefore, AruC could maintain the balance between L-arginine and L-glutamate and fine-tune the glutamate pool. The upregulation of lysine and methionine transporters indicated that zinc-stressed *P. putida* exhibited more demand for some other amino acids. Simply obtaining amino acids from the environment provided a more energy-efficient way than provoking their de novo synthesis.

**Table 5:** Expression ratios of genes involved in basic metabolism^a^.

***P. putida* KT2440 ORF^b^**	**Gene name**	**Fold change^c^**			**Annotation**
		**0.2 mmol L^−1^**	**1.5 mmol L^−1^**	**2.5 mmol L^−1^**	
**AMINO ACIDS METABOLISM**					
PP_0220	*metNB*	–^d^	**7.50** ± **1.30**	-	Methionine ABC transporter ATP-binding protein
PP_0269		–	**42.33** ± **2.45**	**61.33** ± **3.95**	Glutamate synthase large subunit
PP_0372	*aruC*	–	**95.5** ± **0.12**	**137.5** ± **6.21**	Acetylornithine aminotransferase
PP_0699		–	**4.93** ± **0.04**	**4.8** ± **0.12**	LysE family transporter
PP_0999	*arcC*	–	0.27 ± 0.03	**0.23** ± **0.09**	Carbamate kinase
PP_1110		–	–	**5.81** ± **1.28**	Serine acetyltransferase
PP_1400	*kgtP*	–	0.27 ± 0.01	**0.19** ± **0.04**	Alpha-ketoglutarate permease
PP_2453	*ansB*	–	**7.07** ± **0.45**	3.53 ± 0.33	Glutaminase-asparaginase
PP_3593		–	**4.17** ± **0.23**	**9.00** ± **0.24**	Amino acid ABC transporter substrate-binding protein
PP_3596	*amaD*	–	2.0 ± 0.35	**4.20** ± **0.57**	D-lysine oxidase
PP_5033	*hutU*	–	–	**4.50** ± **1.15**	Urocanate hydratase
**CARBON METABOLISM AND ENERGY GENERATION**					
PP_0104	*ctaD*	–	**0.18** ± **0.03**	0.36 ± 0.01	Cytochrome c oxidase subunit I
PP_0105		–	**0.17** ± **0.02**	0.36 ± 0.07	Cytochrome c oxidase assembly protein
PP_0110		–	**0.24** ± **0.03**	0.38 ± 0.00	CyoE-like protoheme IX farnesyltransferase
PP_0154	*scpC*	–	**6.78** ± **0.35**	**7.22** ± **2.33**	Propionyl-CoA:succinate CoA transferase
PP_0328	*fdhA*	–	**0.20** ± **0.01**	**0.21** ± **0.01**	Formaldehyde dehydrogenase
PP_0545	*aldB*1	–	**0.17** ± **0.01**	**0.20** ± **0.08**	Aldehyde dehydrogenase
PP_0557	*acoR*	–	**0.24** ± **0.00**	0.26 ± 0.06	Acetoin catabolism regulatory protein
PP_0763		–	0.38 ± 0.01	**0.13** ± **0.06**	Medium-chain-fatty acid CoA ligase
PP_0944	*fumC*1	–	2.2 ± 0.07	**4.50** ± **0.77**	Class II fumarate hydratase
PP_1016	*gtsB*	–	**0.11** ± **0.07**	**0.08** ± **0.00**	Mannose/glucose ABC transporter permease
PP_1017	*gtsC*	–	**0.12** ± **0.00**	**0.11** ± **0.01**	Mannose/glucose ABC transporter permease
PP_1018	*gtsD*	–	**0.17** ± **0.01**	**0.13** ± **0.01**	Mannose/glucose ABC transporter ATP binding protein
PP_2379		–	**7.51** ± **0.07**	**4.88** ± **0.13**	Cytochrome oxidase biogenesis protein
PP_2988		–	**4.5** ± **1.59**	**11.50** ± **2.49**	Alcohol dehydrogenase
PP_3122	*atoA*	–	2.5 ± 0.06	**4.09** ± **0.90**	3-Oxoacid CoA-transferase subunit A
PP_3332		–	**6.83** ± **1.53**	**6.00** ± **1.36**	Cytochrome c-type protein
PP_4116	*aceA*	–	3.25 ± 0.16	**11.00** ± **0.28**	Isocitrate lyase
PP_4251	*ccoO*1	–	**5.47** ± **1.05**	–	cbb3-type cytochrome c oxidase subunit
PP_4252	*ccoQ1*	–	**5.40** ± **1.12**	–	cbb3-type cytochrome c oxidase subunit
PP_4253	*ccoP*1	1.11	**5.20** ± **0.07**	–	cbb3-type cytochrome c oxidase subunit
PP_4297	*gcl*	–	**4.75** ± **1.21**	**17.00** ± **2.69**	Glyoxylate carboligase
PP_4487	*acsA*1	–	**5.56** ± **0.31**	**13.00** ± **2.44**	Acetyl-CoA synthetase
**PROTEIN METABOLISM**					
PP_1360	*groS*	–	**6.10** ± **0.50**	3.52 ± 0.64	Co-chaperonin GroES
PP_1361	*groL*	–	**5.85** ± **0.56**	3.29 ± 0.88	Chaperonin GroEL
PP_1982	*ibpA*	–	**5.31** ± **0.67**	**14.48** ± **2.03**	Small heat shock protein IbpA
PP_4179	*htpG*	–	**5.88** ± **0.56**	**4.13** ± **1.49**	Chaperone protein HtpG
PP_4727	*dnaK*	–	**5.25** ± **0.52**	**4.14** ± **1.28**	Chaperone protein DnaK
PP_4728	*grpE*	–	**5.60** ± **0.04**	**4.05** ± **1.13**	Heat shock protein GrpE
PP_5000	*hslV*	–	**7.82** ± **0.54**	**7.07** ± **0.09**	ATP-dependent HslVU protease peptidase subunit
PP_5001	*hslU*	–	**6.77** ± **0.41**	**5.41** ± **1.65**	Protease HslVU ATPase subunit
**NUCLEIC ACID METABOLISM**					
PP_0034		–	**182.00** ± **8.95**	–	Ribonuclease
PP_0353		–	**52.33** ± **0.47**	**98.00** ± **3.30**	Exonuclease
PP_1116		–	**4.00** ± **0.23**	–	Resolvase family site-specific recombinase
PP_2454	*rbsB*	–	**0.21** ± **0.07**	0.29 ± 0.10	Ribose ABC transporter,periplasmic ribose-binding subunite
PP_4033	*rnz*	–	**0.18** ± **0.02**	**0.24** ± **0.00**	Ribonuclease Z
PP_4034	*hyuC*	–	**0.14** ± **0.01**	**0.19** ± **0.02**	Bifunctional N-carbamoyl-beta-alanine amidohydrolase/allantoine amidohydrolase
PP_4035	*pydP*	–	**0.14** ± **0.01**	**0.19** ± **0.02**	NCS1 family transporter PydP
PP_4037	*pydX*	–	**0.16** ± **0.01**	0.28 ± 0.08	NADP-dependent dihydropyrimidine dehydrogenase subunit
PP_4038	*pydA*	–	**0.16** ± **0.00**	0.31 ± 0.06	NADP-dependent dihydropyrimidine dehydrogenase subunit PreA

a Expression levels of genes in P. putida KT2440 were measured using RNA-seq, as described in section Materials and methods. The data are means of three replicates. If a gene was strongly changed more than 4-fold at one experimental condition, the changes at other conditions are also shown.

b Gene name, number, and annotation are from the Pseudomonas genome project (Winsor et al., 2016).

c Fold change ≥4 or ≤ 0.25 was illustrated in bold.

d *Dashes mean that the transcription was not significantly changed (Fold change ≤ 2 or ≥0.5, or P_adj_ > 0.01)*.

In addition to amino acid metabolism genes, transcriptional changes in the genes involved in carbon source metabolism and energy generation were also observed. The only carbon source presented in the CDM medium was glucose. Metabolic flux analysis revealed that glucose is mainly assimilated through Enter–Doudoroff pathway in *P. putida*, generating most of the pyruvate; then the flow of carbon is directed toward the citric acid cycle (TCA cycle) under aerobic conditions (Sudarsan et al., 2014). After zinc treatment, most of the genes involved in the Enter–Doudoroff pathway and TCA cycle appeared to be stable, only the fumarate hydratase encoding gene (*fumC*1) was upregulated under the high zinc stress. *aceA* was another gene that only upregulated by 2.5 mmol L^−1^ zinc, which encodes an enzyme in the glyoxylate shunt (GS) that catalyzes the cleavage of isocitrate to succinate and glyoxylate. The GS in bacterial physiology has traditionally been associated with the demand for gluconeogenesis, as induced by carbon-source limitation. However, growing evidence suggests that oxidative stress activate the GS (Ahn et al., 2016). Under usual conditions, cells produce ROS as by-products of their aerobic respiration or nutrient oxidation (Hobman et al., 2007). GS bypasses two NADH-generation steps in TCA cycle, which diminishes the electrons flux funneled into respiration. Therefore, activation of GS limits the further exacerbation of oxidative stress induced by zinc. According to Alhasawi et al. (2014), *P. fluorescence* tends to use another way to reduced NADH generation under zinc stress. The activities of TCA cycle enzymes, such as isocitrate dehydrogenase, α-ketoglutarate dehydrogenase, and malate dehydrogenase, were markedly diminished. To survive in changing environmental conditions, *P. putida* KT2440 evolved branched respiratory chains containing five different terminal oxidases with different affinities for oxygen and capabilty to pump protons (Morales et al., 2006). Regulation of these oxidases is quite complex and has not been completely unraveled (Follonier et al., 2013). Although the cytochrome Cbb3-1 oxidase (PP_4251, PP_4252, and PP_4253) was remarkably upregulated and the Aa3-type (PP_0104, and PP_0105) was downregulated under the intermediate zinc stress, the underlying mechanism remains unknown.

Another group of genes whose transcription was altered in zinc-treated samples was that of genes involved in protein folding and degradation. Six chaperone encoding genes (*groES, groEL, ibpA, dnaK, grpE*, and *htpG*) were upregulated after 1.5 and 2.5 mmol L^−1^ zinc treatment, suggesting a greater protein folding efficiency. An ATP-dependent protease belonging to Clp family (HslUV) was also induced. Clp proteases comprise proteolytic and regulatory components (Manara et al., 2012); some of them play a decisive role in determining the density of proteins under both normal and stressed conditions (Gómez-Sagasti et al., 2015). The upregulation of *hslUV* might be required in response to zinc stress to recycle unnecessary proteins rapidly or to remove proteins denatured by zinc ions. Pertaining to nucleic acid metabolism, the recycling of nucleotide or base was also enhanced because an exonuclease (PP_0353) was greatly upregulated and a nucleobase cation symporter (PP_4035) as well as the genes (PP_4037, and PP_4038) responsible for pyrimidine degradation were strongly downregulated.

### RT-qPCR validation

**Figure 4:**
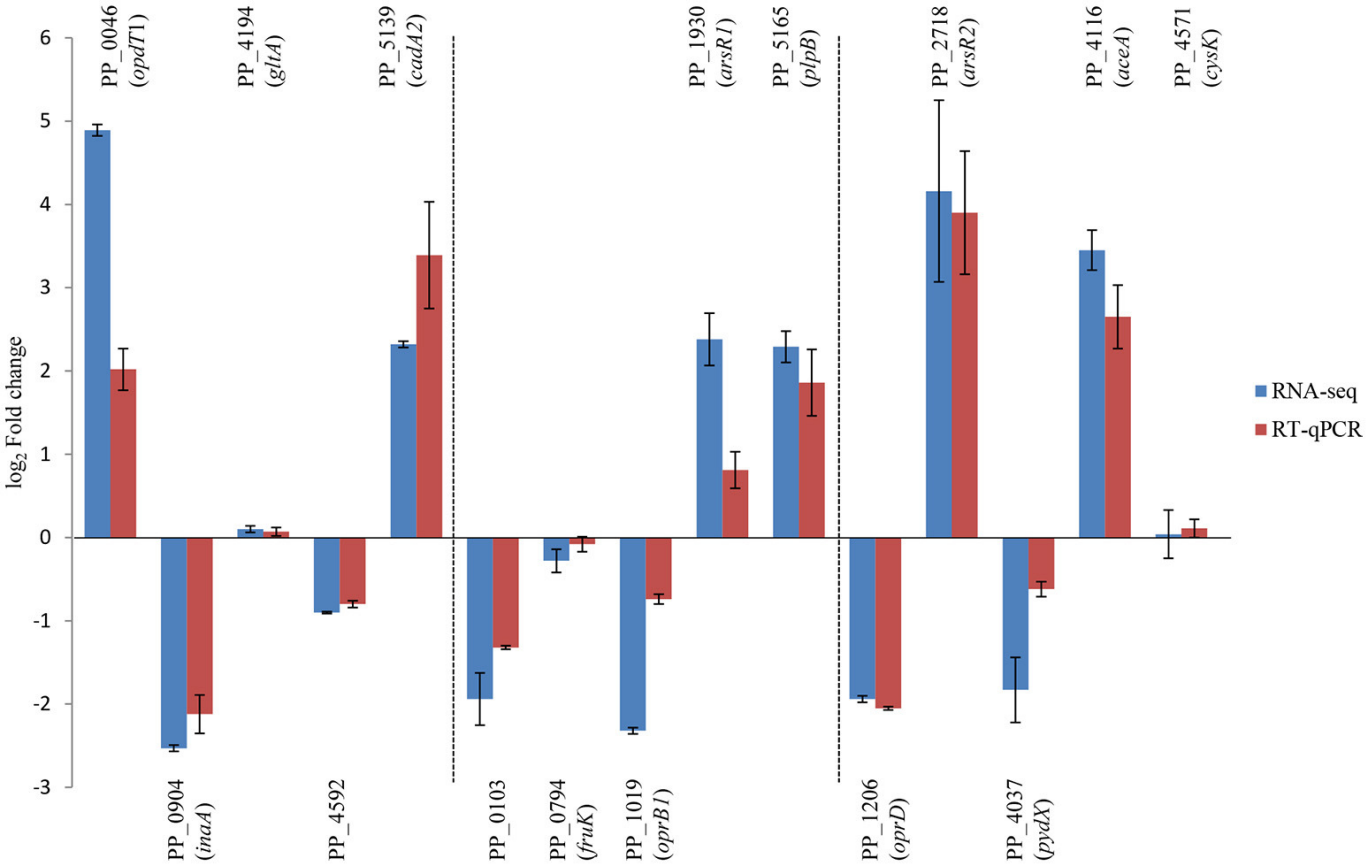
Validation of RNA-seq results by RT-qPCR. Rectangles represent transcription changes of selected genes as measured by RNA-seq and RT-qPCR. Bars illustrate standard deviations of three replicates.

**Figure 5:**
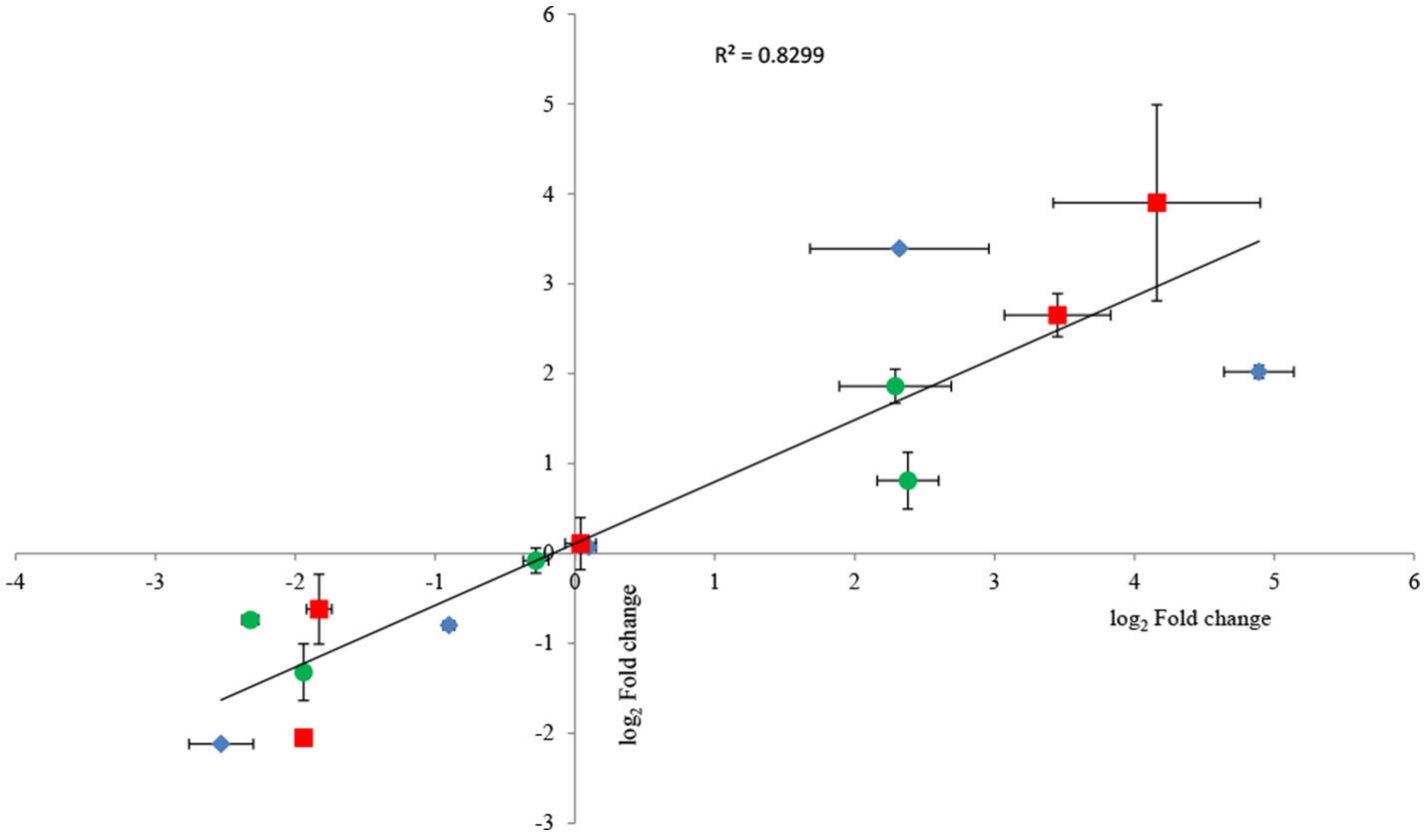
Correlation plot between fold changes of selected genes observed in RNA-seq and their corresponding fold-change values in RT-qPCR analysis. Horizontal bars represent standard deviations for RT-qPCR data, and vertical bars illustrate standard deviations of RNA-seq results. Three replicates were carried out for both RNA-seq and RT-qPCR analysis. As all groups followed the normal distribution, Pearson's correlation coefficient was calculated to determine the level of correlation. The symbols ♦, •, and ■ represent the genes selected from the samples under the low, intermediate, and high zinc stress, respectively.

To confirm RNA-seq data, the expression levels of 15 genes were examined via RT-qPCR in triplicate. For all 15 genes, the same expression trend was detected under RT-qPCR and RNAseq analyses (Figure 4). Additionally, the observed fold changes for each gene were moderately correlated (*r*^2^ = 0.83, Figure 5). Therefore, the RT-qPCR results confirm the accuracy and consistency of the RNA-seq data. Moreover, several genes previously known to be induced or repressed by zinc further validate the capability of RNA-seq experiments to identify candidate genes responding to toxic concentrations of external zinc.

### Comparative analysis of gene expression patterns

In 2016, Mumm et al. (2016) analyzed the response of *P. putida* and its *colR*-deficient strain to zinc excess at the whole-proteome level. Although different culture conditions could cause different cellular responses (Kim et al., 2013), we still found quite a few similarities in gene expression patterns. Both proteomic and transcriptomic analysis revealed that the most upregulated genes under zinc stress were the genes responsible for metal or multidrug efflux systems, whereas the most downregulated genes were some porin-encoding genes. Besides, cell envelop was fine-tuned with the regulation of lipoprotein synthesis. The induction of oxidative response genes and genes involved in Fe-S cluster synthesis were not observed at the proteome level, which indicated that 0.6 mmol L^−1^ zinc ions in LB medium only generated a relatively low level of zinc stress.

*E. coli* is another bacterial species whose response to zinc has been intensively analyzed. Genome analysis revealed that *P. putida* evolved a greater number of genes responsible for metal resistance than *E. coli*. Our results and the data reported by Mumm et al. (2016) indicated that some other differences were observed at transcriptomic and proteomic levels. *E. coli* and *P. putida* both enhanced the synthesis of lipopolysaccharides or lipoproteins that constitute outer membrane, however, only *P. putida* greatly altered the expression of porins. In addition, these two strains may use different ways to chelate cellular free zinc ions. In *E. coli*, positive regulation of cysteine synthesis operon was a remarkable feature in both nutrient-rich and nutrient-limited medium containing low concentrations of zinc (Hobman et al., 2007). Large amounts of cysteine residues were needed to play a role in transient trapping of excess free zinc ions prior to export (Yamamoto and Ishihama, 2005; Etesami, 2018). In *P. putida*, upregulation of cysteine synthesis was not observed under low or intermediate zinc stress; only serine acetyltransferase which catalyzes the first step that convert serine to cysteine was moderately upregulated at a really high zinc concentration.

## Conclusion

The transcriptional response of *P. putida* KT2440 to elevated concentrations of zinc was analyzed in this study. After comparing the different RNA-seq datasets obtained, 213 genes were found to be transcriptionally changed by more than 4-fold. Zinc ions have no redox activity under normal conditions. Many previous researches also confirmed that zinc excess did not cause cellular oxidative stress. However, this is true only for the bacteria under low zinc concentrations. As the concentration increased, zinc ions clearly induced the generation of ROS in *P. putida* cells, as evidenced by the upregulation of alkylhydroperoxide reductase and ferredoxin-NADPH reductase. At the highest zinc dose, the central carbon source metabolism pathways (glycolysis and TCA cycle) of *P. putida* were very stable, whereas the synthesis of glutamate and the recycle use of protein and nucleotide were activated. GS shunt was also upregulated at the highest zinc concentration, which limited the further exacerbation of oxidative stress by decreasing the electron flux funneled into respiration chain. Although zinc-stressed *P. putida* cells tended to use different terminal enzymes in their branched respiration chain, the underlying mechanism is poorly understood and merits further investigation.

## Data availability statement

The RNA-seq datasets for this study can be found in the NCBI short read archive database under the Bioproject accession number PRJNA450701.

## Author contributions

PL and XC conceived the experiment. JP and LM performed the experiment. PL wrote the manuscript.

### Conflict of interest statement

The authors declare that the research was conducted in the absence of any commercial or financial relationships that could be construed as a potential conflict of interest.

## Abbreviations

CDM: cation-defined medium used in this study
RT-qPCR: reverse transcription quantitative PCR
nMDS: non-metric multidimensional scaling
ROS: reactive oxygen species
GS: glyoxylate shunt
TCA cycle: citric acid cycle.
