# Supplementary material for: Comparative Transcriptome Analysis of Pseudomonas putida KT2440 Revealed Its Response Mechanisms to Elevated Levels of Zinc Stress
Source: Front Microbiol. 2018 Jul 24;9:1669. doi: 10.3389/fmicb.2018.01669 (PMC6066579; doi:10.3389/fmicb.2018.01669)
Supplement: Supplementary file 1 [file Table_1.DOCX]

Table S1 Selected ORFs and their corresponding primer pairs used in RT-qPCR analysis

| ORFs | Sequence | Fragment Size (bp) |
| --- | --- | --- |
| PP_0191  (Rsd/AlgQ) | 5‘- GAGATCAACCCACGGATCAACGACA -3’ | 131 |
|  | 5‘- TCATGCAACAACCCGCCCAAT -3’ |  |
| PP_0904 | 5‘- GCGAGTGGGTCGAGGAGCCTAAC -3’ | 172 |
|  | 5‘- TTCAGCGCATCGAGTTCACGCAACA -3’ |  |
| PP_4592 | 5‘- TCAAGGGCTGTCGTCGCTGTTTCGC -3’ | 160 |
|  | 5‘- GGCCGCATCACGCTTGGCATCTT -3’ |  |
| PP_5139 | 5‘- CCTGTGGAGAAGGGGGTGGGTGA -3’ | 193 |
|  | 5‘- CCGGGGTGTAGATGCGCGAAAAACG -3’ |  |
| PP_0046 | 5‘- CACCGCCCCCACTTACAACAGT -3’ | 130 |
|  | 5‘- AAAGGCATCAACGCCAAAGCCAACG -3’ |  |
| PP_5165 | 5‘- CGGCCTGATTACCTTGAAAGACCCG -3’ | 234 |
|  | 5‘- ACCAGGAAGTTCACGTAAGGCGAGT -3’ |  |
| PP_0103 | 5‘- CCCTTCCTGATTCTGGTGGTGAT -3’ | 133 |
|  | 5‘- GGCCCAGGTATTTGTAGTGCCATTT -3’ |  |
| PP_1930 | 5‘- GCCCGTGAAGGCGAACTCTGTGTCT -3’ | 208 |
|  | 5‘- ATTCCTGGTTGGCATCGACCACTCC -3’ |  |
| PP_1019 | 5‘- CTGCCGAGGCTTTTTCCAGCGAATC -3’ | 119 |
|  | 5‘- GCCAGCCACCTCACCCACATAATCG -3’ |  |
| PP_2718 | 5‘- AGCCAACCCAAGATCAGTCG -3’ | 109 |
|  | 5‘- TTCTTCAGCCGAAGGGCATT-3’ |  |
| PP_4116 | 5‘- GTGCAACCTGAGCACACCTTTG -3’ | 114 |
|  | 5‘- GCCCATGCAGTTGACGAAATCTTTG -3’ |  |
| PP_1206 | 5‘- CCTACAACATCGGCGCTCACACCTT -3’ | 160 |
|  | 5‘- CGAGCCTGCCAGGATTTCTCGTCTT -3’ |  |
| PP_4037 | 5‘- CCCAGACGGCTCATCTGCACAGC -3’ | 127 |
|  | 5‘- CCACCGAATACATCCGCGAGCTACG -3’ |  |
| PP_0794 | 5‘- CCTGCGCTGGATATCACCAT -3’ | 113 |
|  | 5‘-AGTACCTGGGCAACGTTCAG-3’ |  |
| PP_1290 | 5‘-AAAGCTCGAAGGCACGTTCT-3’ | 123 |
|  | 5‘-TACTCCCACCCCTGGATACC-3’ |  |
| PP_4571 | 5‘-GCGAAGAACTCAAGCCAAGC-3’ | 120 |
|  | 5‘-CCTTGGACTCTTCGTCGGTC-3’ |  |
