# Supplementary material for: Comparative Transcriptome Analysis of Pseudomonas putida KT2440 Revealed Its Response Mechanisms to Elevated Levels of Zinc Stress
Source: Front Microbiol. 2018 Jul 24;9:1669. doi: 10.3389/fmicb.2018.01669 (PMC6066579; doi:10.3389/fmicb.2018.01669)
Supplement: Supplementary file 2 [file Table_2.DOCX]

Table S2 The number of reads obtained from each sample

| Sample | Replicate | Total clean reads (million) | Total mapped reads (%) |
| --- | --- | --- | --- |
|  | 1 | 15.83 | 86.44 |
| Control | 2 | 11.65 | 86.49 |
|  | 3 | 13.52 | 94.21 |
|  | 1 | 12.14 | 73.73 |
| Cells treated with 0.2 mmol L^-1^ zinc | 2 | 14.77 | 73.45 |
|  | 3 | 13.86 | 82.74 |
|  | 1 | 13.51 | 86.21 |
| Cells treated with 1.5 mmol L^-1^ zinc | 2 | 13.38 | 87.61 |
|  | 3 | 12.44 | 92.98 |
|  | 1 | 12.52 | 78.18 |
| Cells treated with 2.5 mmol L^-1^ zinc | 2 | 11.38 | 71.12 |
|  | 3 | 12.17 | 86.89 |
