# Supplementary material for: Comparative Transcriptome Analysis of Pseudomonas putida KT2440 Revealed Its Response Mechanisms to Elevated Levels of Zinc Stress
Source: Front Microbiol. 2018 Jul 24;9:1669. doi: 10.3389/fmicb.2018.01669 (PMC6066579; doi:10.3389/fmicb.2018.01669)
Supplement: Supplementary file 3 [file Table_3.DOCX]

Table S3 Predicted noncoding RNAs whose transcription were strongly influenced by zinc stress (more than fourfold)

| Transcription Start | Transcription Stop | Fold change | | |
| --- | --- | --- | --- | --- |
|  |  | 0.2 mmol L^-1^ zinc | 1.5 mmol L^-1^ zinc | 2.5 mmol L^-1^ zinc |
| 30143 | 30394 |  | 26.50 | 16.90 |
| 32305 | 32352 |  | 208.33 |  |
| 161710 | 161749 |  |  | 18.73 |
| 161727 | 161749 |  | 10.51 |  |
| 500581 | 500614 |  | 0.08 |  |
| 501652 | 501709 |  | 0.068 |  |
| 501658 | 501701 |  |  | 0.04 |
| 978504 | 978547 |  |  | 5.15 |
| 1531983 | 1532018 |  |  | 4.65 |
| 2295720 | 2295757 |  |  | 11.36 |
| 4298567 | 4298596 |  |  | 4.84 |
| 4298575 | 4298621 | 5.21 |  |  |
| 4545813 | 4545846 |  |  | 0.18 |
| 4653199 | 4653424 |  |  | 10.69 |
| 5117724 | 5117743 |  |  | 4.01 |
| 5179354 | 5179512 |  | 19.32 | 52.11 |
| 5338286 | 5338614 |  | 0.08 | 0.06 |
